# Anti-Coronavirus Efficiency and Redox-Modulating Capacity of Polyphenol-Rich Extracts from Traditional Bulgarian Medicinal Plants

**DOI:** 10.3390/life12071088

**Published:** 2022-07-20

**Authors:** Neli Vilhelmova-Ilieva, Zdravka Petrova, Almira Georgieva, Elina Tzvetanova, Madlena Trepechova, Milka Mileva

**Affiliations:** 1 The Stephan Angeloff Institute of Microbiology, Bulgarian Academy of Sciences, 26 Georgi Bonchev, 1113 Sofia, Bulgaria; nelivili@gmail.com (N.V.-I.); zdr.z1971@abv.bg (Z.P.); al.georgieva@inb.bas.bg (A.G.); elinaroum@yahoo.com (E.T.); madi_trepechova@yahoo.com (M.T.); 2 Institute of Morphology, Pathology and Anthropology with Museum, Bulgarian Academy of Sciences, 25 Georgi Bonchev, 1113 Sofia, Bulgaria; 3 Institute of Neurobiology, Bulgarian Academy of Sciences, 23 Acad. G. Bontchev St., 1113 Sofia, Bulgaria

**Keywords:** natural extracts, coronavirus infection, virucidal activity, viral adsorption, antiradical and metal-chelating capacity

## Abstract

**Background:** The use of various herbal therapists as part of traditional medicine in different parts of the world, including Bulgaria, is due to the knowledge accumulated over the centuries by people about their valuable biological activities. In this study, we investigate extracts from widely used Bulgarian medicinal plants for their ability to prevent the coronavirus infection of cells by testing different mechanisms of antiviral protection, their polyphenol content, and redox-modulating capacity. **Methods:** The influence on the stage of viral adsorption, the inhibition of extracellular virions, and the protective effect on uninfected cells of the plant’s extracts were reported by the end-point dilution method, and virus titer (in Δ lgs) was determined as compared to the untreated controls. The total content of polyphenols and flavonoids was also determined. We tested the antioxidant power of the extracts by their ability to inhibit the generation of superoxide anionic radicals and to scavenge DPPH radicals. We determined their iron-reducing, copper-reducing, and metal-chelating antioxidant powers. **Results:** Most of the extracts tested suppress the extracellular virions of HCov. They also inhibit the stage of viral adsorption to the host cell to varying degrees and have a protective effect on healthy cells before being subjected to viral invasion. The examined extracts contained significant levels of polyphenols and quercetin-like flavonoids and showed remarkable antioxidant, radical, and redox-modulating effects. **Conclusions:** All of these 13 extracts from Bulgarian medicinal plants tested can act as antioxidants and antiviral and symptomatic drugs for the management of coronavirus infection.

## 1. Introduction

Natural products have been used for millennia for the treatment of human diseases. The diversity of plant species and the knowledge accumulated by people over the centuries for their proper use as herbal medicines makes possible the existence of traditional medicine in different parts of the world. Of the approximately 250,000 flowering plant species described, between 50,000 and 70,000 are known to be used in traditional and modern medicine worldwide [1]. Recently, we have witnessed a resurgence of global interest in herbal medicines. More and more researchers are turning to the study of herbal products in healthcare.

Bulgaria is a country with a small territory and with great potential in terms of herbal drugs. The Bulgarian flora is rich in medicinal plants— it contains more than 4300 plant species, and over 500 are rare or endemic to the countries of the Balkan area. Only 750–770 of all the plant species are medicinal plants, and most of them are wild-growing and occur freely in nature. People collect and use these species that are known for their medicinal properties, mostly in the treatment of viral and bacterial infections.

It is widely known that viral and bacterial infections are the most common causes of disease in human society. A wide range of a specific and nonspecific chemotherapeutics have been developed to treat sundry viral infections, but their use is followed by side effects, which are followed by the development of different degrees of resistant strains that are not susceptible to the therapy used [2].

In retrospect, herbal medicinal plants have been used for thousands of years as a medicinal approach for the treatment of various viral illnesses. Products derived from medicinal plants are more easily absorbed by the body because of their natural origin and show fewer side effects because they are structurally close to normal cellular components [3,4,5]. The potential for the development of resistant strains to natural antiviral agents is significantly reduced compared to chemotherapeutics due to their complicated chemical structure and too often because of their multistage mode of action.

Many plant sources are also used in cooking as spices and food additives, and so they are involved indirectly in human metabolism through the daily food intake, manifesting their healing effect.

For example, medicinal plants such as *O. basilici*, *A. sativumi*, *T. vulgaris*, *R. canina*, *A. officinalis* and others act in a similar way [6,7,8]. There is evidence that *A. officinalis* is used purposefully when coughing, *A. annua* is used in “fever” conditions, and in various forms of inflammation *A. hippocastani*, *A. officinalis*, *H. perforati*, *M. chamomillae*, *O. basilicum*, *P. lanceolata*, *P. reptans*, *R. caninae*, *T. vulgaris* and others are recommended [9,10,11,12,13,14,15,16]. *H. perforata*, *R. canina*, and *T. vulgaris* have a neuroprotective and calming effect, and extracts of *A. annua*, *G. glabra*, *T. vulgaris*, *P. reptans*, *R. canina*, and *P. lanceolata* have an antispasmodic effect [11,15,16,17,18].

Many extracts of medicinal plants have been studied for their antitumor activity against various forms of cancer (*A. annuae*, *T. vulgaris*, *P. reptans*, *R. caninae*, *P. lanceolata*, etc.) [11,15,16,17,18]. Very good antifungal, antibacterial, and antiviral activity were shown in extracts of *P. reptans*, *O. basilicum*, *G. glabra*, and *A. Annuae* [6,16,17,19].

The combination of honey and *O. basilici* against Gram-positive and Gram-negative bacteria shows a significant antibacterial effect [20]. *P. lanceolata* has been shown to have anti-inflammatory activity in *E. coli* infection, probably due to the high content of polyphenols in the extract. [21]. *R. caninae* has an inhibitory effect on multidrug-resistant bacterial strains, including *S. aureus* [22]. Extracts of *H. perforati*, *A. annua*, *P. reptans*, *T. vulgaris*, *G. glabra* and others also show antibacterial activity [9,12,13,16,19].

The activity of some extracts on the replication of various viruses, as well as on the viability of extracellular virions, was also studied. *A. hippocastani* extract showed virucidal and antiviral activity against enveloped HSV-1, VSV, and dengue viruses in vitro, as well as virucidal activity against RSV and antiviral activity in vivo [23]. *A. sativum* is widely used for the prevention and treatment of many viral diseases in humans, animals, and plants by inhibiting viral RNA-pol, reverse transcriptase, and DNA synthesis [24]. *S. nigra* shows activity against influenza virus by blocking viral glycoproteins, as well as against *Infectious bronchitis virus* (IBV)—pathogenic chicken coronavirus [25]. Antiviral activity against RNA viruses, including CoVs—feline infectious peritonitis (FIP)—feline coronavirus, is also exhibited in *T. vulgaris* extract [26]. Extracts of *T. vulgaris* and *O. basilici* show anti-HIV-1 activity, and the same extracts also show anti-HSV-1 activity [6,7]. *G. glabra* extract also demonstrates antiherpes activity [19].

Some of the antibacterial and antiviral activities are due to the immunomodulatory action of the extracts in the multicellular organism [7,10,24,25].

Extracts, essential oils, or tinctures of different parts of plants can suppress the symptoms of some illnesses due to the scavenging of free radicals in a different way or to inhibit their generation. From a chemical point of view, a free radical is a relatively stable structure that contains one or more unpaired electrons. It has the ability with other molecules, either by taking it away from another molecule to increase stability or by donating its unpaired electron to another molecule starting, and a chain process is initiated; one radical gives rise to another radical.

In a biological context, free radicals relate to reactive oxygen species, which are formed at mitochondrial respiration [27]; they are the results of a variety of enzymatic reactions such as NADPH oxidases, xanthine oxidase, nitric oxide synthase, lipoxygenases, and cyclooxygenases; they may be products of ionizing and UV radiation, or by the metabolism of a wide range of drugs and xenobiotics [28]. Stationary (physiological) levels of ROS are intrinsic to the normal functioning of cells, fulfilling the functions of cell signaling and homeostasis [29]. On the other hand, in pathological conditions when they are produced in excess or when cellular defenses are not able to metabolize them, oxidative stress damage occurs. At this moment, antioxidants take their place—(i) endogenous, which are enzymatic or nonenzymatic produced by the body; or (ii) exogenous, which are taken through food, as supplements, or in combination with conventional drugs. As antioxidants can reduce the risk of inflammatory disease development by counterbalancing an excess of reactive species, they are vital for human health [30].

It has been reported that extracts of *A. annua*, *G. glabra*, *A. hippocastani*, *A. officinalis*, *H. perforati*, *M. chamomillae*, *O. basilici*, *P. lanceolata*, *P. reptans*, *R. canina*, *T. vulgaris*, etc. are rich in polyphenols and especially quercetin-like flavonoids, so they are able to control the symptoms of the so-called “oxidative stress diseases”, i.e., inflammations and respiratory virus infections [7,8,11,15,16,17,19,31,32,33].

The last three years showed humanity a significant example of the dangers and seriousness of the prevention, treatment, and control of viral diseases via the COVID-19 pandemic situation. The search for new approaches to control the pathogenesis of this virus infection, with extremely diverse symptoms, led us to study the antiviral activities of a panel of Bulgarian medicinal plants that are widely used to relieve symptoms often found in patients with COVID-19 (Table 1).

As it turns out, the therapeutic effects of medicinal plants depend first on their ingredients and their solubility in various solvents (water, alcohol, fat, etc.). In turn, their solubility determines the drug forms for their preparation and administration—infusions, decocts, tinctures, syrups, oils, tablets, ointments, and balms or directly on the skin. These herbal products are known among doctors and pharmacists as phytopharmaceuticals that are prepared from different plant parts (roots, stems, leaves, flowers, fruits) [3].

The aim of the present study was to investigate extracts from some widely used Bulgarian medicinal plants: (i) for their ability to prevent the coronavirus infection of cells by testing different mechanisms of antiviral protection; (ii) to be analyzed their polyphenol content, and (iii) to be studied their redox-modulating capacity.

## 2. Materials and Methods

### 2.1. Antiviral Investigations

This work was supported by the National Science Fund at the Republic Bulgaria approved by Research Grant No. KП-06-ДK1/3 of the Ministry of Education and Science.

Antiviral research conducted at the Stephan Angeloff Institute of Microbiology, Bulgarian Academy of Sciences, which is a member of the World Organization of Institutes of the Louis Pasteur Network, meets all the necessary requirements for good practices in virology and microbiology, with safety level 2. The researchers certify their responsibilities.

#### 2.1.1. Host Cell Culture

Diploid cell line MCR-5 derived from normal lung tissue was purchased from the American Type Culture Collection (ATCC). Cells were incubated at 37 °C in the presence of 5% CO_2_ using Eagle’s Minimum Essential Medium (Capricorn Scientific GmbH, Ebsdorfergrund, Germany), supplemented with 10% Fetal bovine serum and (Gibco BRL, St. Louis, MO, USA) and 100 IU penicillin and 0.1 mg streptomycin/mL (Sigma-Aldrich, St. Louis, MO, USA).

#### 2.1.2. Virus

Human coronavirus (HCoV)—229E strain (ATCC: VR-740) strain was replicated in monolayer MRC-5 cells in Eagle’s Minimum Essential Medium supplemented with 2% Fetal bovine serum, 100 U/mL penicillin, and 100 μg/mL streptomycin. The cells were incubated with the virus for 5 days at 35 °C and 5% CO_2_ and then lysed by double freezing and thawing. The virus was titrated by the method of Reed and Muench. Viral aliquots were stored at −80 °C.

#### 2.1.3. Researched Plant Material

A total of 13 dry water-ethanol (15 °C) extracts of Bulgarian medicinal plants were produced and provided by Extractpharma Ltd.,Sofia, Bulgaria, one of the leading companies in Bulgaria for production and trade in food supplements, herbal products, and liquid and dry extracts of medicinal plants, Sofia, Bulgaria (Table 2).

#### 2.1.4. Production of Dry Extract

The standardized herbal mixture (according to the recipe) was extracted in an extraction installation on the principle of countercurrent, by performing a classic solid–liquid extraction. The extractant was water-ethanol 15 °C. The extraction was carried out at atmospheric pressure and temperature of 40 °C for 18 h. The resulting liquid herbal extract was concentrated to a certain percentage of dry matter, depending on the type of herb, and dried in dryers: (1) vacuum-drying plant and (2) powder-drying plant to obtain a dry extract. The extracted herb was not reused.

#### 2.1.5. Cytotoxicity Assay

Confluent monolayer cell culture in 96-well plates (Costar^®^, Corning Inc., Kennebunk, ME, USA) was treated with 0.1 mL/well support medium containing decreasing concentrations of tested extracts. The cells were incubated at 37 °C and 5% CO_2_ for 120 h. After microscopic evaluation, the medium containing the test extracts was removed, and the cells were washed and incubated with neutral red at 37 °C for 3 h. After incubation, the neutral-red dye was removed, and the cells were washed with PBS and 0.15 mL/well desorbing solution (1% glacial acetic acid and 49% ethanol in distilled water) was added. The optical density (OD) of each well was read at 540 nm in a microplate reader (Biotek Organon, West Chester, PA, USA). A total of 50% cytotoxic concentration (CC_50_) was defined as the concentration of the material that reduces cell viability by 50% compared to untreated controls. Each sample was tested in triplicate with four wells for cell culture on a test sample.

The maximum tolerable concentration (MTC) of the extracts was also determined, which is the concentration at which they do not affect the cell monolayer, and in the samples, it looks like the cells in the control sample (untreated with extract).

#### 2.1.6. Virucidal Assay

Samples of 1 mL containing HCoV (with 10^5^ cell-culture infectious dose 50 (CCID_50_)) and tested extract in its maximal tolerable concentration (MTC) were contacted in a 1:1 ratio and subsequently stored at room temperature for different time intervals (15, 30, 60, 90, and 120 min). Then, the residual infectious virus content in each sample was determined by the end-point dilution method, and Δlgs as compared to the untreated controls was evaluated.

#### 2.1.7. Effect on Viral Adsorption

Some 24-well plates containing monolayer cell culture from MRC-5 cells were pre-cooled to 4 °C and inoculated with 10^4^ CCID_50_ of human coronavirus. In parallel, they are treated with tested extracts at their maximum tolerable concentration (MTC) and incubated at 4 °C for the time of virus adsorption. At various time intervals (15, 30, 60, 90 and 120 min), the cells were washed with PBS to remove both the compound and the unattached virus, then coated with support medium and incubated at 35 °C for 120 h. After freezing and thawing three times, the infectious viral titer of each sample was determined by the final dilution method. Δlg was determined compared to the viral control (untreated with the compounds). Each sample was prepared in four replicates.

#### 2.1.8. Pretreatment of Healthy Cells

Cell monolayers grown in 24-well cell-culture plates (CELLSTAR, Greiner Bio-One) were treated for different time intervals—15, 30, 60, 90, and 120 min at maximum tolerable concentration (MTC) of extracts in maintenance medium (1 mL/well). After the above time intervals, the compounds were removed, and the cells were washed with phosphate buffered saline (PBS) and inoculated with human coronavirus (1000 CCID_50_ in 1 mL/well). After 120 min of adsorption, the nonadsorbed virus was removed, and the cells were coated with a maintenance medium. Samples were incubated at 35 °C for 120 h and, after freezing and thawing three times, infectious virus titers were determined by the final dilution method. Δlg was determined compared to the viral control (untreated with the compounds). Each sample was prepared in four replicates.

### 2.2. Redox-Modulating Capacity

All reagents used for the preparation of reaction mixtures, namely potassium salts, thiobarbituric acid, riboflavin, methionine, nitro- blue tetrazolium, and 1,1-diphenyl-2-picryl-hydrazyl (DPPH), 2,4,6-tripyrydil-s-triazine, hydrochloric acid, and ferric chloride were obtained from Sigma-Aldrich (Darmstadt, Germany).

#### 2.2.1. Determination of the Total Flavonoid Content

The total flavonoid content (TFC) was determined using the aluminum chloride colorimetric method reported by Gouveia and Castilho (2011) [94], with slight modifications: 0.30 mL of methanol, 0.02 mL of 10% AlCl_3_, and 0.56 mL of distilled water were combined with 0.5 mL of appropriately diluted samples. For 30 min, the vials were incubated at room temperature. TFC was estimated as micrograms of quercetin equivalents (QE) per gram of plant extract using a 415 nm absorbance.

#### 2.2.2. Determination of the Total Polyphenol Content

The Folin–Ciocalteu assay was used to assess the total phenol content (TPC) of the extracts [95]. To 0.150 mL samples in disposable test tubes were added 0.750 mL Folin–reagent Ciocalteu’s (diluted 1:10 with deionized water) and 0.600 mL sodium carbonate (7.5 percent). The tubes were incubated for 10 min at 50 °C, and the absorbance was measured at 760 nanometers. TPC was calculated as microgram gallic acid equivalents (GAE) per gram of plant extract.

#### 2.2.3. Superoxide Anion Radical Generating System (^●^O_2_^−^)

We used the method of Beauchamp and Fridovich (1971) [96] for the photochemically generation of superoxide anion radicals (^●^O_2_^−^), in a medium containing 50 mM potassium phosphate buffer, pH 7.8; 1.17 × 10^−6^ M riboflavin; 0.2 mM methionine; 2 × 10^−5^ M KCN and 5.6 × 10^−5^ M nitro-blue tetrazolium (NBT). The NBT reduction by ^●^O_2_^−^ to a blue formazan product, in the absence (control) and the presence of different concentrations (5; 2.5; 1.25; 0.625; 0.3125 mg/mL) of the tested substances, was measured at 560 nm. The antioxidant capacity of the plant extracts was expressed as a half-maximal inhibitory concentration IC_50_.

#### 2.2.4. DPPH Radical-Scavenging Assay

The measurement of the DPPH radical-scavenging activity was performed according to Brand-Williams (1995) [97]. The tested substances in different concentrations as follows (5; 2.5; 1.25; 0.625; 0.3125; 0.156; 0.0781; 0.04 mg/mL) reacted with the stable DPPH in methanol solution. The reduction of DPPH^●^ to DPPHH led to changes in color (from deep violet to light yellow), which was read at 517 nm after 30 min incubation at room temperature. A mixture of methanol and sample served as blank, and a mixture of methanol and DPPH radical solution served as control. The scavenging activity percentage (AA%) was determined as follows:

AA% = 100 − [(Abs (sample) − Abs (blank) × 100)/Abs (control)]

#### 2.2.5. Ferric-Reducing Antioxidant Power (FRAP)

The FRAP assay was performed according to Benzie and Strain (1996) [98] with some modifications using the following solutions: (1) 30 mM acetate buffer at pH 3.6; (2) 1 mM TPTZ (2,4,6-Tri(2-pyridyl)-s-triazine) in 40 mM HCl; (3) 1.5 mM FeCl_3_. Thus, prepared solutions were mixed in the following ratio: 10 parts (1): 1 part (2): 20 parts (3). To a 50 mL sample put in a disposable test tube was added 1.5 mL of reaction mixture: blank–reaction mixture + 50 mL of deionized H_2_O. The method is based on the reduction in Fe (III) ion if the sample contains a reductant (antioxidant) to Fe (II) at low pH. The colorless Fe (III)-TPTZ complex is transformed into the blue Fe (II)-TPZ complex after incubation for 4′ at 37 °C. Absorption was measured at 593 nm. The results were expressed as µmol Trolox equivalents per g of extract.

#### 2.2.6. Cupric-Reducing Antioxidant Capacity (CUPRAC)

The cupric-reducing antioxidant capacity (CUPRAC) was performed according to Apak et al. (2004) [99] with some adaptation. In the ratio of 1:1:1, the following solutions were mixed: 10 mM CuCl_2_ in ddH_2_O, 1.0 M ammonium acetate buffer pH 7.0, and 7.5 mM neocuproin (NC) in 96% ethanol. In 96-well plates, 80 µL was pipetted from different concentrations (5; 2.5; 1.25; 0.625; 0.3125 mg/mL) of the tested substances, then 220 µL of the reaction mixture was added. After incubation at 50 °C for 20 min, absorbance was measured at 450 nm against a blank (220 µL reaction mixture and 80 µL H_2_O). The standard curve was prepared with Trolox at various concentrations ranging from 0.1 to 1.0 mM, and the results were expressed as µM Trolox equivalent/1 g extract.

#### 2.2.7. Iron-Chelating Power

The reaction of iron (II) ions with ferrozine produces a pink complex with a maximum absorption wavelength of 562 nm. The addition of a sample containing a chelating agent lowers the observed absorbance. Procedure: 0.2 mL of sample solution was mixed with 0.74 mL of 0.1 M sodium/acetate buffer (pH 5.23) and 0.02 mL of 2 mM FeSO_4_ in 0.2 M HCl. After 10–15 s, 0.04 mL of 5 mM ferrozine was added. After 10 min of staying in the dark, the absorbance was measured. The formula for determining the Fe (II) chelating capability of the tested material is:

Activity (%) = 100 (Ac − As)/(Ac),

where Ac is the absorbance of the blank probe, instead containing all of the sample −200 µL sodium/acetate buffer. As is the absorbance of the sample solution [100]. The Fe (II)-chelating capability is expressed as mM EDTA equivalent per 1 g extract.

### 2.3. Statistical Analysis

Data on cytotoxicity were analyzed statistically. The values of CC_50_ were presented as means ± SD. All measurements of redox-modulating activities were made in triplicate and the data in graphics were presented as a means ± standard deviation.

## 3. Results

### 3.1. Anticoronavirus Acitvities

#### 3.1.1. Cytotoxicity of the Thirteen Extracts against the MRC-5 Cell Line

The cytotoxicity of the thirteen extracts against the MRC-5 cell line was determined. Cytotoxic concentrations of 50% (CC_50_) and maximum tolerable concentrations (MTC) of the extracts were determined. When comparing the cytotoxicity of the studied extracts, it was noticed that the lowest cytotoxicity was shown by *A. officinalis* extract with values for CC_50_ = 2100 µg/mL. The cytotoxicities of extracts of *S. nigra* (CC_50_ = 1750 µg/mL), *A. sativum* and *G. glabra* (CC_50_ = 1700 µg/mL), *P. reptans* (CC_50_ = 1600 µg/mL), and *P. lanceolata* were also relatively low (CC_50_ = 1560 µg/mL). For all extracts, the highest cytotoxicity was shown in *M. chamomilla* extract with CC_50_ = 820 µg/mL and *H. perforatum* (CC_50_ = 830 µg/mL). The maximum tolerable concentrations of the extracts required for many of the following experiments were also determined (Table 2).

#### 3.1.2. Virucidal Activity of Extracts against Human Coronavirus Virions

The next step of our investigation was to monitor whether the extracts we studied could protect healthy cells from viral invasion. The subjects of the study were MRC-5 cells and human coronavirus. Three model systems were used. In the first, the direct effect of the extracts on extracellular virions was determined. Four of the extracts showed virucidal activity in the first study time interval (15 min). The most pronounced effect was observed in the extract of *T. vulgaris* (Δlg = 2.5), followed by the extracts of *M. chamomillae* (Δlg = 2.25), *A. sativum* (Δlg = 2.0), and *P. reptans* (Δlg = 1.75), and this effect remains significant at all studied time intervals. In *A. sativum* and *M. chamonillae*, after 90 min of exposure the inhibitory effect is slightly enhanced. At a 30 min exposure time, *G. glabra* extract (Δlg = 1.75) also showed significant activity, which increased with increasing exposure time. At a contact time of 90 min, the extract of *O. basilicum* significantly reduced the viral titer by Δlg = 2.25, and the extract of *A. hippocastani* caused a significant decrease in viral yield after exposure of 120 min. 

Extracts of *P. lanceolata*, *H. perforatum*, *A. officinalis* (Δlg = 1.5), and *A. annua* (Δlg = 1.25) showed minimal effects. *S. nigra* extract showed no effect on extracellular viral particles (Table 3).

#### 3.1.3. Effect of the Extracts on the Adsorption Step of HCoV Virions to Host Cells

In the second experimental setup, we traced the effect of the extracts on the adsorption step of HCoV virions to host cells. The effect of the extracts was reported at various time intervals. In the first-time interval (15 min) studied, only four of the extracts significantly inhibited viral adsorption, the most notable being the effect of *T. vulgaris* extract, which reduced viral yield by Δlg = 2.5. The activity of *M. chamomilla* extract (Δlg = 2.25), *A. sativum* (Δlg = 2.0) and *P. reptans* (Δlg = 1.75) was also strong. The *S. nigra* extract showed no activity at an exposure time of 15 min, while the other extracts inhibited the adsorption step of HCoV on sensitive cells, but to a lesser extent. As the exposure time increased, the initially reported effect of 15 min was either maintained or slightly enhanced. The extract from *T. vulgaris* at 120 min retained the same activity together with that from chamomile extract (Δlg = 2.5). *A. sativum* slightly enhanced its activity by lowering the viral titer by Δlg = 2.25. The activity of extracts from *G. glabra*, *A. annua* and *A. officinalis* (Δlg = 2.0), as well as *P. reptans* and *O. basilicum* (Δlg = 1.75) is also significant. Extracts of *R. canina*, *A. hippocastani*, *P. lanceolata* and *H. perforatum* weakly inhibited the adsorption step of HCoV, and *S. nigra* extract did not affect this step at any of the observed time intervals (Table 4).

#### 3.1.4. Protective Effect of the Studied Extracts on Pretreated Healthy Cells

In the next experimental model that we used to monitor the protective effect of the studied extracts, we pretreated healthy cells with the extracts at their maximum tolerable concentration followed by HCoV infection. Almost all tested extracts had varying degrees of protection on uninfected cells, reducing their susceptibility to attachment, entry, and production of new viral progeny in the host cell. The strongest protection for 15 min of treatment was demonstrated by garlic and rosehip extracts (Δlg = 2.5). With close activity were extracts from *P. reptans* (Δlg = 2.25), *A. officinalis* and *O. basilicum* (Δlg = 2.0). The effect of the extract from *A. hippocastani*, *G. glabra*, and *H. perforati* was also significant (Δlg = 1.75). When treated for longer intervals, the protective result of the extracts increased. In the last reported time interval—120 min—the most pronounced was the protective effect of the extract from *A. hippocastani*, which almost completely prevented subsequent coronavirus infection by lowering the viral titer by Δlg = 4.5. Also remarkable was the protection of extracts from *S. nigra* and *M. chamomilla* (Δlg = 3.5), as well as extracts from *A. sativum* and *P. lanceolata* (Δlg = 3.25). The protective activity of *P. reptans* (Δlg = 2.75), *G. glabra*, *H. perforatum*, *A. officinalis* and *O. basilicum* (Δlg = 2.0) is also significant. At this stage of protecting the cell from viral infection, extracts of *T. vulgaris* and *A. annua* did not show significant activity (Table 5).

### 3.2. Content of Polyphenols, Total Polyphenols, and Redox-Modulating Properties

All tested extracts contained significant amounts of polyphenols comparable to gallic acid and quercetin—similar flavonoids—and showed significant antioxidant, radical scavenging, and redox-modulating effects (Table 6). The extracts of *Ocimum basilicum* and *Hypericum perforatum* proved to be the richest in polyphenols in quantities corresponding to 7.2–7.3 mg gallic acid/g extract, followed by *Sambucus nigra* and *Rosa canina* L. They also showed the best iron-reducing effect, together with *Thymus vulgaris*.

*Ocimum basilicum* extract has proven to be an excellent radical cleansing, chelating metal, iron, and copper-reducing agent. It showed the best properties as an acceptor of DPPH radicals and the lowest IC_50_ as an inhibitor of superoxide anion radical generation. Our results revealed another also highly active redox modulator, the extract of *Rosa canina* L., followed by *Sambucus nigra*, *Potentilla reptans*, *Thymus vulgaris*, and *Artemisia annua*. As can be seen in Table 6, there is a strong relationship between the content of polyphenols, flavonoids, and the manifested redox-modulating capacity of the studied extracts. It has been shown that the healing properties of plant extracts are mainly due to the presence of these compounds, which have the ideal molecular structure of excellent antioxidants, redox modulators, and medicinal substances [101,102].

## 4. Discussion

To the best of our knowledge, this is the first systematic study on polyphenol analysis, antiviral activity, and redox-modulating capacity activity of these Bulgarian representatives of plants. All products derived from natural sources are a mix of secondary metabolites (alkaloids, flavonoids, organic acids, tannins, polysaccharides, lipids, saponins, glycosides, anthraquinones, terpenes, etc.) [6,11,14,15]. The content of these products is due to the therapeutic effect of herbalists: anticancer, antioxidant, antidiabetic, immunosuppressive, antifungal, anti-inflammatory, antimalarial, antibacterial, antipyretic, antidiabetic, insecticidal, antiviral and others [17,31,33,101,102,103,104,105,106]. Plant bioactive components can be used as therapeutics with new targets other than those targeted by existing synthetic therapists. In Bulgaria, as well as worldwide, there is an abundance of medicinal plants, but their use as potential antivirals is poorly studied. For example, *A. annua* affects some viral diseases and the artemisinin contained in it shows SARS-CoV-1 and 2 activities [13]. Electron microscopy has shown that the polyphenol contained in *S. nigra* compromises the viral envelopes of infectious bronchitis virus (IBV), pathogenic chicken coronavirus. It is assumed that this is the probable mechanism of action of the extract of this plant.

It is possible that the mechanism of action of the extracts we studied is similar, which explains their virucidal activity and inhibition of stage of viral adsorption. Most likely, the components contained in them affect the structure of the viral envelope, thereby impairing the ability of the virus to attach and enter the host cell. On the other hand, natural metabolites apparently could bind to components of the cell membrane—receptors and other structures necessary for the recognition and penetration of the virus—without adversely affecting the cell. In this way, the cell becomes unsusceptible to the virus. Such a mechanism of action can explain the protective effect that we observed in pretreated cells, as well as the inhibition of the viral adsorption step.

New mutating viruses require new pharmacological approaches, especially during the ongoing COVID-19 pandemic and due to the lack of efficient treatment. Recently, special attention has been paid to studying plants rich in polyphenols that can be efficient against coronavirus infections [6,11,14,15,101,102,103,104,105,106].

Finding new products to inhibit the penetration and replication of pathogens from the coronavirus family, especially the spread of SARS-CoV-2 (severe acute respiratory syndrome) is of great importance for human health just now. In this case, studied plant extracts from Bulgarian flora that are rich in quercetin-like compounds, gallic acid, and other polyphenols and inhibit the growth cycle of viruses are welcome in therapeutic schemes for treatment and prevention.

It has been proven that polyphenols protect from viral infections, and in case of infection, support the healing process by various mechanisms: (i) they block the entry into the host cells; (i) inhibit the multiplication of the virus; (iii) support anti-inflammatory functions and the human body’s defense by modulating immune regulation and inhibiting cytokine storms; (iv) they also inhibit pro-inflammatory cytokines and model cellular immunity, so they act as immunomodulators; (v) support anti-inflammatory functions and the human body’s defense by modulating immune regulation and inhibiting cytokine storms; (vi) act as free radical scavengers [101,102,103,104,105,106].

Both COVID-19 and all viral infections have been reported to cause oxidative stress in infected tissues, following a possible cytokine storm, blood clotting, and exacerbation of hypoxia—a life-threatening systemic inflammatory syndrome involving elevated levels of circulating cytokines—as well as iron overload [107]. Therefore, antioxidant therapy and the removal of excess iron from the body with special drugs can be useful in the prevention of oxidative stress symptoms.

As a rule, antioxidants can have different functions depending on the type of radical species produced and of the target molecule protected. They can be divided into three subgroups according to their defense mechanisms: (i) preventive, (ii) radical scavenger, and (iii) enzyme-repairing.

Plant polyphenols are secondary metabolites with strong antioxidant capacities. Plants synthesize them for their own defense against oxidative stress, but these compounds retain the ability to act as antioxidants ex planta and thus largely contribute to the biological properties of plant-derived drugs and supplements [108]. In this context, the characterization of polyphenols is an important point for characterizing the antioxidant properties of medicinal drugs. Flavonoids, as well as many other plant polyphenols, possess a chemical structure ideal for free-radical scavenging [109]. Their antioxidant properties include reactivity to a variety of reactive oxygen species, as well as metal chelating.

One of the most dangerous effects of overproduction of ROS is ferroptosis. It is a type of iron-dependent, oxidative cell death that can be caused by a variety of factors. Ferroptosis is different from apoptosis but is also the result of dysfunction of antioxidant defense, leading to loss of cellular redox homeostasis [110,111].

An increase in ROS in the presence of iron ions has been shown to lead to ferroptosis. There are data showing that the overgeneration of ROS and ferroptosis can be controlled by treatment with deferoxamine as an iron chelator [112].

The mechanisms of FRAP and CUPRAC methods are based on a single electron transfer. The reducing power of FRAP cannot detect antioxidants that act by radical quenching (H transfer). The FRAP method is based on the reduction of Fe (III) to Fe (II), and the CUPRAC method is based on the reduction of Cu (II) to Cu (I) [113]. When iron (III) and copper (II) are in a “free” form they can catalyze the production of highly toxic hydroxyl radicals by dint of one electron donating, so the revocation of one electron is critical importance for the normal cell’s functions [114].

Antioxidants that could chelate and reduce iron (III) ions are potential candidates for controlling ferroptosis and its destructive effects on healthy cells. The high metal-reduction capacity in our study suggests that extracts from Bulgarian medicinal plants can be the first line of defense against copper toxicity and serve as a copper chelator by sequestering the metal in a non-redox-active form.

The analysis of oxidative-stress biomarkers and components of the antioxidative defense system in various forms of disease induced by COVID-19 infection, as well as in different aspects of pathogenicity during the disease, could indicate the role of oxidative stress and can suggest timely intervention using various antioxidants. Correlations of oxidative stress with the immune response could provide insight into the interaction between the immune system and oxidative damage.

This mechanism of antioxidant activity is beneficial to live organisms due to the prevention of oxidative damage to the cellular membranes and is essential for cell survival. According to our investigations, the studied 13 plant extracts might perform an essential detoxification function against ions of copper and iron. This function would be beneficial for maintaining the metal homeostasis and protecting the function of cellular structures against the damaging effects of reactive oxygen species.

## 5. Conclusions

In this investigation, we screened herbal medicinal plants from Bulgarian flora as potential anti-coronavirus medications. Based on the literature databases, more of them are described by modes of action as symptomatic drugs on the viral infections. We added some data to these findings by the testing different mechanisms of antiviral protection against coronavirus infection by (i) cell cytotoxicity, based on maximum tolerable concentration of extracts; (ii) inhibition of viral attachment and penetration in the host cells on the data of their residual infectious virus content; and (iii) virucidal assay, based on decreasing of virus titer of the samples.

The study of the mechanism of antioxidant activity of drugs and substances supplemented in condition of the diseases associated with viral pathogenesis is beneficial to live organisms due to the prevention of oxidative damage to the cellular membranes, and because it is essential for cell survival. According to our results, the 13 studied plant extracts, along with the typical healing benefits, might perform an essential detoxification function against ions of copper and iron. This function would be beneficial for maintaining metal homeostasis and protecting the function of cellular structures against the damaging effects of reactive oxygen species. Plant extracts from this investigation can act as antioxidants and antiviral and symptomatic drugs for the management of coronavirus infection. We hope this finding will help researchers and clinicians to identify the source of appropriate antiviral drugs from plants in combating coronaviruses, and ultimately, save millions of affected human lives.

## Figures and Tables

**Table 1:** Clearly expressed healing antiviral and symptomatic effects in the context of anti-coronavirus symptoms of the Bulgarian medicinal plants tested.

Plant Species/Drug	Control of the Respiratory Infections					
	Cold	Bronchitis	Cough	Pain	Fever	Viral Diseases
***S. nigra***	[34]	[35]	[36]	[37,38]	[34]	[34,36,39,40]
***A. sativum***	[41]	[42]	[43]	[44]	[45]	[24]
***P. reptans***	[46]	-	-	-	[47]	[47]
***R. canina***	-	[48]	[48]	[49]	[50]	[51]
***M. chamomilla***	[52]	[53]	[54]	-	[54]	[52,55]
***A. hippocastani***	[56]	-	[57]	[58]	[59]	[57]
***G. glabra***	[60]	[61]	[62]	[63]	[64]	[65,66]
***P. lanceolata***	[67]	[68]	[69]	[70]	[71]	[67]
***H. perforatum***	-	[72]	[73]	[74]	[73]	[73,75]
***T. vulgaris***	[76]	[7]	[76]	[77]	[76]	[78,79]
***A. annua***	[80]	[81]	[80]	[82]	[83]	[84,85]
***A. officinalis***	[86]	[86]	[86]	[87]	[88]	[55]
***O. basilicum***	[89]	[90]	[90]	[91]	[92]	[89,93]

**Table 2:** Plant species and cytotoxicity of the extracts.

Plant Species		Area of the Collected Material	Cytotoxicity (µg/mL)	
			CC_50_	MTC
***Sambucus nigra*** **(elderberry)**	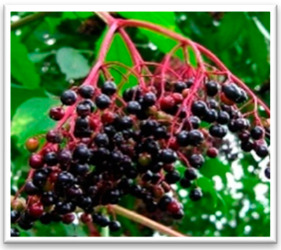	Fruit	1750 ± 35.2	1000
***Allium sativum*** **(garlic)**	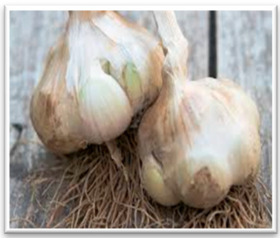	Root	1700 ± 33.2	1000
***Potentilla reptans*** **(Creeping cinquefoil)**	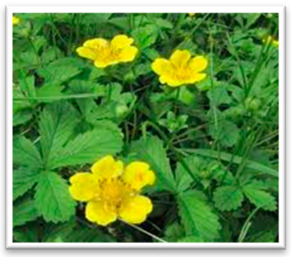	Steam	1600 ± 13.6	200
***Rosa canina* L.** **(rosehip)**	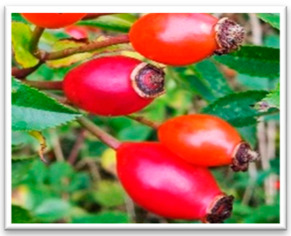	Fruit	1480 ± 32.4	1000
***Matricaria chamomilla* L.** **(chamomile)**	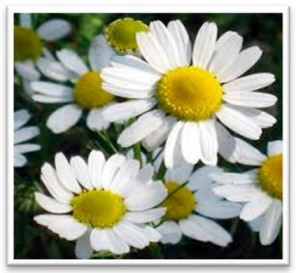	Flower	820 ± 8.5	1000
***Aesculus*** ***hippocastanum*** **(horse chestnut)**	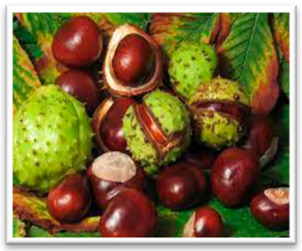	Seed	1220 ± 23.6	800
***Glycyrrhiza glabra* L.** **(licorice)**	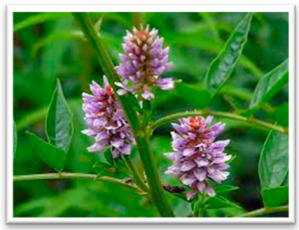	Root	1700 ± 42.7	1000
***Plantago lanceolata*** **(Ribwort plantain)**	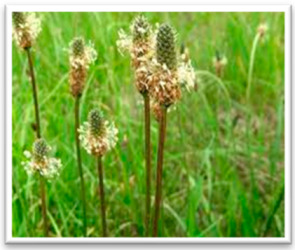	Stem	1560 ± 41.3	1000
***Hypericum perforatum*** **(St. John’s Wort)**	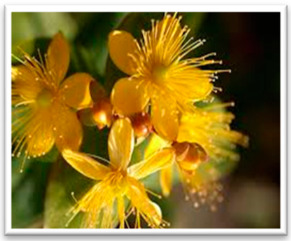	Steam	830 ± 12.4	500
***Thymus vulgaris*** **(thyme)**	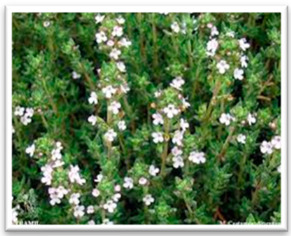	Steam	880 ± 18.4	320
***Artemisia annua*** **(sweet wormwood)**	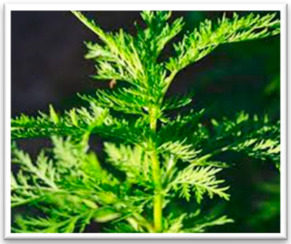	Steam	1150 ± 36.1	320
***Althaea officinalis*** **(Marsh Mallow)**	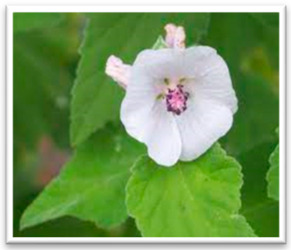	Steam	2100 ± 42.1	1500
***Ocimum basilicum*** **(basil)**	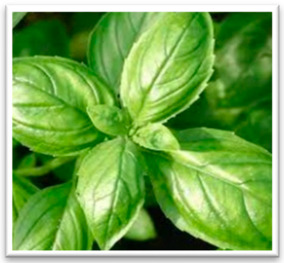	Stem	1250 ± 19.3	1000

**Table 3:** Virucidal activity of extracts against human coronavirus virions.

Extract	Δlg				
	15 min	30 min	60 min	90 min	120 min
***Sambucus nigra***	0	0	0.5	0.5	0.5
***Allium sativum***	2.0	2.0	2.0	2.25	2.25
***Potentilla reptans***	1.75	1.75	1.75	1.75	1.75
***Rosa canina* L.**	1.0	1.0	1.25	1.25	1.25
***Matricaria chamomilla* L.**	2.25	2.25	2.25	2.5	2.5
***Aesculus hippocastanum***	1.0	1.0	1.25	1.5	1.75
***Glycyrrhiza glabra* L.**	1.5	1.75	1.75	2.0	2.0
***Plantago lanceolata***	1.5	1.5	1.5	1.5	1.5
***Hypericum perforatum***	1.5	1.5	1.5	1.5	1.5
***Thymus vulgaris***	2.5	2.5	2.5	2.5	2.5
***Artemisia annua***	1.25	1.25	1.25	1.25	1.25
***Althaea officinalis***	1.25	1.25	1.5	1.5	1.5
***Ocimum basilicum***	1.25	1.25	1.5	2.25	2.25

**Table 4:** Influence of the extracts on the stage of adsorption of HCoV to sensitive MRC-5 cells.

Extract	Δlg				
	15 min	30 min	60 min	90 min	120 min
***Sambucus nigra***	0	0	0.5	0.5	0.5
***Allium sativum***	2.0	2.0	2.0	2.25	2.25
***Potentilla reptans***	1.75	1.75	1.75	1.75	1.75
***Rosa canina* L.**	1.0	1.0	1.25	1.25	1.25
***Matricaria chamomilla* L.**	2.25	2.25	2.25	2.5	2.5
***Aesculus hippocastanum***	1.0	1.0	1.25	1.25	1.25
***Glycyrrhiza glabra* L.**	1.5	1.75	1.75	2.0	2.0
***Plantago lanceolata***	1.5	1.5	1.5	1.5	1.5
***Hypericum perforatum***	1.5	1.5	1.5	1.5	1.5
***Thymus vulgaris***	2.5	2.5	2.5	2.5	2.5
***Artemisia annua***	1.25	1.25	1.5	1.5	2.0
***Althaea officinalis***	1.25	1.25	1.5	1.5	2.0
***Ocimum basilicum***	1.25	1.25	1.5	1.5	1.75

**Table 5:** Protective effect of pretreatment of extracts on healthy MRC-5 cells and subsequent HCoV infection.

Extract	Δlg				
	15 min	30 min	60 min	90 min	120 min
***Sambucus nigra***	1.5	2.0	3.25	3.25	3.5
***Allium sativum***	2.5	2.5	3.0	3.25	3.25
***Potentilla reptans***	2.25	2.25	2.5	2.75	2.75
***Rosa canina* L.**	2.5	2.5	2.5	2.5	2.5
***Matricaria chamomilla* L.**	1.0	2.5	3.25	3.25	3.5
***Aesculus hippocastanum***	1.75	1.75	4.25	4.5	4.5
***Glycyrrhiza glabra* L.**	1.75	1.75	2.0	2.0	2.0
***Plantago lanceolata***	1.5	1.5	3.25	3.25	3.25
***Hypericum perforatum***	1.75	1.75	2.0	2.0	2.0
***Thymus vulgaris***	0.5	0.5	0.5	1.0	1.0
***Artemisia annua***	0.5	0.5	0.5	1.0	1.0
***Althaea officinalis***	2.0	2.0	2.0	2.0	2.0
***Ocimum basilicum***	2.0	2.0	2.0	2.0	2.0

**Table 6:** Total polyphenols, total flavonoids content, radical-scavenging, and metal-chelating activities of extracts.

Plant Species	Total Polyphenols * [mg Gallic Acid/g Extract]	Total Flavonoids ** [mg Quercetin/g Extract]	FRAP ^#^	CUPRAC ^##^	Fe (II) Chelating ^++^	DPPH Scavenging Activity, %	Inhibition of Superoxide Generation, IC_50_ [mg/mL]
***Sambucus nigra***	5.92 ± 0.23	0.98 ± 0.05	8.20 ± 0.13	126.80 ± 5.87	1.50 ± 0.03	53.93	2.85
***Allium sativum***	0.36 ± 0.05	0.12 ± 0.00	-	2.23 ± 0.85	1.07 ± 0.05	5.53	3.02
***Potentilla reptans***	4.14 ± 0.05	0.84 ± 0.11	7.32 ± 0.77	116.79 ± 13.99	1.56 ± 0.4	50.19	1.72
***Rosa canina* L.**	5.69 ± 1.64	0.44 ± 0.07	14.44 ± 1.46	217.72 ± 9.35	1.83 ± 0.14	48.48	1.12
***Matricaria chamomilla* L.**	2.07 ± 0.04	0.58 ± 0.10	2.07 ± 0.30	30.59 ± 0.00	1.22 ± 0.07	25.14	1.64
***Aesculus hippocastanum***	2.05 ± 0.10	0.79 ± 0.11	1.41 ± 0.35	110.31 ± 2.00	2.43 ± 0.61	16.26	27.35
***Glycyrrhiza glabra* L.**	3.26 ± 0.06	1.81 ± 0.09	1.65 ± 0.16	135.09 ± 0.00	2.43 ± 0.61	16.26	10.76
***Plantago lanceolata***	1.77 ± 0.02	0.40 ± 0.10	3.74 ± 0.20	108.22 ± 0.00	0.63 ± 0.12	25.76	0.66
***Hypericum perforatum***	7.24 ± 0.23	1.60 ± 0.09	12.00 ± 1.43	172.93 ± 16.11	1.89 ± 0.11	64.36	1.05
***Thymus vulgaris***	4.87 ± 0.08	1.04 ± 0.14	9.70 ± 0.77	141.88 ± 16.95	1.09 ± 0.13	52.53	7.33
***Artemisia annua***	3.76 ± 0.20	1.05 ± 0.12	6.22 ± 0.54	46.62 ± 11.55	1.26 ± 0.18	48.64	1.1
***Althaea officinalis***	1.45 ± 0.29	0.36 ± 0.10	1.46 ± 0.70	14.75 ± 2.05	1.17 ± 0.28	8.79	9.49
***Ocimum basilicum***	7.32 ± 0.25	1.01 ± 0.15	14.24 ± 1.59	235.49 ± 22.50	0.98 ± 0.10	64.36	0.19

**Legend:** * The total polyphenolic content is calculated from the gallic acid calibration curve and is expressed as µg gallic acid/mg extract; ** The total flavonoid content was calculated from a quercetin calibration curve and was expressed as µg quercetin/mg extract. ^#^ Fe (III)-reducing activity is calculated from a Trolox calibration curve and is expressed as µM Trolox equivalent/1 g extract. ^##^ Cu (II) reducing activity is calculated from a Trolox calibration curve and is expressed as µM Trolox equivalent/1 g extract. ^++^ Fe-chelating activity is calculated from the EDTA calibration curve and is expressed as mM EDTA equivalent/1 g extract; DPPH-capture activity is calculated at sample concentration 0.3125 mg/mL extract.

## Data Availability

Not applicable.
